# Vitamin D Attenuates Hepatic Sinusoidal Capillarization in Type 2 Diabetes Mellitus– Metabolic Dysfunction-Associated Fatty Liver Disease via Dual Autophagy Activation and Pyroptosis Suppression in Liver Sinusoidal Endothelial Cells

**DOI:** 10.3390/biomedicines13061459

**Published:** 2025-06-13

**Authors:** Panpan Jiang, Yang Liu, Juxiang Liu, Jinxing Quan

**Affiliations:** 1 The First School of Clinical Medicine, Lanzhou University, Lanzhou 730000, China; lj18394008024@163.com; 2 Department of Endocrinology, Gansu Provincial Hospital, Lanzhou 730000, China; 3 Gansu Provincial Hospital of Traditional Chinese Medicine, Lanzhou 730050, China; liuyddzyx@126.com; 4 Key Laboratory of Endocrine and Metabolic Diseases of Gansu Province, Lanzhou 730000, China

**Keywords:** vitamin D, T2DM, metabolic dysfunction-associated fatty liver disease, autophagy, pyroptosis, LSEC, hepatic sinusoid capillarization

## Abstract

**Background/Objectives:** Metabolic dysfunction-associated fatty liver disease (MAFLD) is closely associated with type 2 diabetes mellitus (T2DM), where T2DM serves as a crucial driving factor for MAFLD progression. While vitamin D (VD) demonstrates protective effects against MAFLD, the underlying mechanisms through which it influences MAFLD-related liver sinusoidal endothelial cell (LSEC) capillarization remain to be elucidated. This study aimed to explore how vitamin D ameliorates LSEC capillarization in T2DM-associated MAFLD. **Methods:** Culture human liver sinusoidal endothelial cells (HLSECs) according to the established protocol. After 1,25(OH)_2_D_3_ intervention in high glucose (HG)-induced HLSECs, determine the changes in liver sinusoidal capillarization-related proteins (LN, PLVAP), autophagy and pyroptosis levels. Observe the changes in cell lipid accumulation and fenestration structures. After adding Bafilomycin A1, MCC950, compound C and rapamycin to HLSECs, explore the therapeutic mechanism of 1,25(OH)_2_D_3_. After supplementing VD to MAFLD model mice, further verify the therapeutic mechanism of VD on MAFLD. **Results:** HG can induce the capillarization and lipid accumulation of HLSEC, increase the level of pyroptosis, and simultaneously reduce the autophagy level. Vitamin D alleviated high-glucose-induced pyroptosis (by suppressing GSDMD/NLRP3) and autophagic inhibition by activating the AMPK-mTOR axis (upregulating p-AMPK and downregulating mTOR), and improved lipid accumulation and hepatic sinusoidal capillarization. In the mouse model of MAFLD, VD supplementation can induce autophagy, inhibit pyroptosis and capillarization, and improve MAFLD. **Conclusions:** These results demonstrate, for the first time, that VD mitigates LSEC dysfunction through dual mechanisms: activating AMPK-dependent autophagy and inhibiting pyroptosis, providing a therapeutic rationale for VD in treating MAFLD-related sinusoidal pathology.

## 1. Introduction

Non-alcoholic fatty liver disease (NAFLD), a prevalent comorbidity of type 2 diabetes mellitus (T2DM), afflicts over 25% of adults globally, posing a substantial public health challenge [1]. This metabolic disorder exhibits progressive pathogenesis, originating as hepatic steatosis and progressing through fibrosis to cirrhosis [2]. Beyond elevating hepatocellular carcinoma risk, NAFLD exacerbates T2DM complications through bidirectional metabolic dysregulation [3]. Indeed, the nomenclature of NAFLD has been updated to metabolic dysfunction-associated fatty liver disease (MAFLD). The new definition of MAFLD refers to hepatic steatosis in addition to one of the following three criteria: overweight/obesity, presence of type 2 diabetes mellitus, or evidence of metabolic dysregulation. MAFLD has been proposed as a more appropriate term, which can better reflect liver diseases associated with known metabolic dysfunctions [4]. In this study, a model of endothelial dysfunction induced by high glucose was adopted, which simulates the core metabolic disorder characteristics of MAFLD. Understanding its pathogenesis is, therefore, critical for developing targeted therapies against this escalating disease burden.

Prior research focused on high-glucose (HG)-induced MAFLD pathogenesis has focused largely on the hepatic parenchymal cells, whereas relatively little is understood of the microvascular aspects of MAFLD. Liver sinusoidal endothelial cells (LSECs) are key determinants of hepatic sinus structure and function, which are responsible for regulating hepatic sinus contraction [5]. LSEC capillarization is an important component of MAFLD development [6,7]. Under physiological conditions, the hepatic sinusoids exhibit discontinuous cell membranes with abundant fenestrae of varying sizes and without any basement membrane underlying the endoderm. These unique structural features allow for compounds to be readily exchanged between the plasma in the hepatic sinusoids and hepatocytes [8]. The endothelium-specific membrane glycoprotein plasmalemma vesicle-associated protein (PLVAP) plays an essential role in hepatic sinusoidal endothelial cell fenestra formation [9]. Hepatic injury results in the aggregation of basement membrane proteins, including the hepatic sinusoidal laminin LN, such that basement membrane formation occurs, together with the absence of PLVAP and the disappearance of fenestrae, thereby leading to capillarization [10,11]. These changes interfere with the ability of plasma compounds to be freely exchanged between the hepatic sinusoids and hepatocytes, contributing to aberrant lipid metabolism and other microenvironmental disturbances that ultimately result in fatty liver onset. Efforts to clarify the molecular and cellular processes that govern hepatic sinusoidal endothelial cell injury and recovery from such damage thus have clear clinical implications.

Pyroptosis, a gasdermin-dependent programmed cell death mechanism, is characterized by DNA fragmentation, membrane perforation, and inflammatory mediator release [12]. In MAFLD pathogenesis, NLRP3 inflammasome activation emerges as a pivotal driver, with clinical evidence showing elevated hepatic mRNA levels of NLRP3, IL-1β, IL-18, and procaspase-1 in patients [13,14,15]. Experimental studies demonstrate that As2O3-induced NLRP3 activation triggers hepatocyte pyroptosis, directly contributing to MAFLD progression [16,17]. While pyroptosis has been increasingly recognized as a critical pathological mechanism in hepatic disorders, its specific role in regulating LSEC capillarization remains unexplored.

Autophagy, an evolutionarily conserved process mediating intracellular component degradation through lysosomal pathways, plays an essential role in maintaining cellular and systemic homeostasis [18]. Emerging as a therapeutic target for MAFLD, this catabolic process demonstrates dual protective mechanisms. First, it directly regulates hepatic lipid metabolism, where its dysregulation contributes to steatosis development [19]. Second, autophagy counteracts key MAFLD pathogenesis factors, including oxidative stress, insulin resistance, and triglyceride accumulation, all of which reciprocally suppress autophagic activity [20]. Autophagy can also play an anti-inflammatory role by coordinating the targeted clearance of intracellular NLRP3 inflammasome activators, structural components, and pro-inflammatory cytokines [21]. Autophagy thus plays a key role in the maintenance and restoration of energy homeostasis, thereby protecting against cell injury.

Vitamin D (VD), a fat-soluble hormone critical for bone mineralization and calcium homeostasis, also modulates hepatic metabolic activity via vitamin D receptor (VDR) signaling [22]. Experimental evidence demonstrates VD3-mediated attenuation of hepatic lipid dysregulation in T2DM models through autophagy activation [23] and VD protection against diet-induced MAFLD via pyroptosis inhibition [24]. While these findings suggest dual regulatory roles of VD in autophagy and pyroptosis, its specific involvement in MAFLD-associated LSEC capillarization remains unexplored. This study aims to explore the roles of pyroptosis and autophagy in the process of capillarization of hepatic sinusoidal endothelial cells in metabolic dysfunction-associated fatty liver disease and to elucidate the protective mechanism of vitamin D exerted by inducing autophagy and inhibiting pyroptosis. We further characterize the molecular crosstalk among autophagy pathways, pyroptotic signaling, and VDR activation within LSECs. Our findings delineate mechanistic connections between VDR signaling, autophagic processes, and pyroptotic pathways in LSECs, advancing understanding of VD’s therapeutic potential in MAFLD management.

## 2. Materials and Methods

### 2.1. Cell Culture

Human LSECs (HLSECs) were obtained from Wuhan Sios Biotechnology Co., Ltd. (Wuhan, China). The cells were cultured in low-glucose (5.5 mM) primary endothelial cell complete culture media (Procell, Wuhan, China) at 37 °C in a humidified atmosphere containing 5% CO_2_ (Thermo Fisher Scientific, Waltham, MA, USA). Cells were cultured in growth media containing 25 mM glucose to represent the HG condition. Cells from passage 5 were used for all experiments.

### 2.2. Cell Viability Assay

HLSEC viability was assessed using the CCK-8 assay. Cells were plated in 96-well plates at 1 × 10^4^ cells/well and incubated overnight at 37 °C. Cells were then pretreated with 1,25(OH)_2_D_3_ (0, 1, 10, 100 nM) for 1 h, followed by 24 h exposure to high glucose (HG, 25 mM). After adding 10 μL CCK-8 solution (Meilunbio, Shanghai, China, MA0218) to each well, plates were incubated at 37 °C for 1 h, and absorbance was measured at 450 nm using a microplate reader (Thermo Fisher Scientific, Shanghai, China).

### 2.3. Cell Navigator Fluorescence Method for Lipid Droplet Detection

HLSECs were plated at 2 × 10^4^ cells/well in 24-well plates and cultured in a 5% CO_2_ humidified atmosphere at 37 °C. After adding 500 µL of Droplite™ working solution (AAT Bioquest, Pleasanton, CA, USA, 22735; prepared in the dark), cells were incubated for 30 min at 37 °C. Subsequently, cells were fixed with 4% paraformaldehyde for 30 min at room temperature, washed three times with PBS, and stained with DAPI (BOSTER, Wuhan, China, AR1176). Fluorescence images were captured using a fluorescence microscope (Olympus, Tokyo, Japan).

### 2.4. Enzyme-Linked Immunosorbent Assay (ELISA)

The levels of IL-1β and IL-18 in HLSEC culture supernatants were measured using ELISA kits according to the manufacturer’s instructions (E-EL-H0149, E-EL-H0253, Elabscience, Wuhan, China).

### 2.5. Western Blotting

Total proteins were extracted from HLSECs or mouse liver tissues, and protein concentrations were determined using a BCA assay kit (BOSTER, Wuhan, China, AR1176). Proteins were resolved on 8–15% SDS-PAGE gels and transferred to PVDF membranes. Membranes were blocked with 5% non-fat milk/TBST for 2 h and then incubated overnight at 4 °C with primary antibodies against NLRP3 (1:1000, GTX133569, GeneTex, San Antonio, TX, USA), Caspase-1 (1:1000, GTX10322, GeneTex), GSDMD (1:1000, ab219800, Abcam), LC3B (1:1000, GTX127375, GeneTex), P62 (1:1000, GTX100685, GeneTex), LN (1:1000, ab277521, Abcam, Waltham, MA, USA), PLVAP (1:1000, ab127554, Abcam), VDR (1:1000, ET1704-09, HUABIO), AMPK (1:1000, GTX50863, GeneTex), p-AMPK (1:1000, GTX52341, GeneTex), mTOR (1:1000, GTX101557, GeneTex), and p-mTOR (1:1000, 5536S, CST, Danvers, MA, USA). After TBST washing, membranes were incubated with HRP-conjugated secondary antibody (1:40,000, HA1001, HUABIO, Hangzhou, China ) for 1 h at room temperature. Signals were detected using an Enhanced ECL kit (Meilunbio, Dalian, China, MA0186) and imaged with a gel documentation system (e-BLOT, Shanghai, China). GAPDH (1:2000, YM3215, Immunoway, Plano, TX, USA) served as the loading control.

### 2.6. Electron Microscopy

Scanning electron microscopy (SEM): HLSECs grown on coverslips were fixed with 2.5% glutaraldehyde, postfixed in 1% osmium tetroxide for 1 h, and dehydrated through an ethanol gradient. Samples were critical-point dried, sputter-coated with gold (JSM-IT700HR, Tokyo, Japan), and imaged. Transmission electron microscopy (TEM): Liver tissues were fixed in 3% glutaraldehyde for 24 h and 1% osmium tetroxide for 1 h, followed by acetone gradient dehydration. Tissues were embedded in Epon 812 resin, sectioned into ultrathin slices (70 nm), and stained with uranyl acetate and Reynolds’ lead citrate. Sections on copper grids were imaged using a TEM (JEOL JEM-1400FLASH, Tokyo, Japan). Under the transmission electron microscope, the fenestrae can be clearly observed to be a direct and permeable channel that extends from the hepatic sinusoid lumen to the intercellular space of hepatocytes. This structural feature of having no diaphragm is one of its important identifying markers.

### 2.7. Adenoviral Transduction

HLSECs in the logarithmic growth phase were seeded in 20 mm glass dishes at 3  ×  10^5^ cells/well. At 30–50% confluence, cells were transduced with 15 μL mRFP-GFP-LC3B adenovirus (Hanheng, Shanghai, China). The medium was refreshed 8 h post-transduction. After 48 h, autophagic flux was assessed using a laser scanning confocal microscope (Zeiss LSM800, Oberkochen, Germany). Early autophagosomes (yellow puncta, mRFP⁺GFP⁺) and late autolysosomes (red puncta, mRFP⁺GFP⁻) were quantified.

### 2.8. Short Hairpin RNA (shRNA) Treatment

VDR-targeting shRNA sequences (designed by Shanghai Genechem Co., Ltd., Shanghai, China): shVDR-96709: 5′-AAGTGCAGAGGAAGCGGGAGA-3′; shVDR-96710: 5′-GTCCAACACACTGCAGACGTA-3′; shVDR-96711: 5′-CCCTGGAGACTTTGACCGGAA-3′; Negative control: 5′-TTCTCCGAACGTGTCACGT-3′. Cells were transfected with shVDR or corresponding negative control constructs following the manufacturer’s protocol. At 72 h post-transfection, VDR knockdown efficiency was validated by Western blotting. shVDR-96710 demonstrated the highest suppression of VDR expression and was selected for further studies.

### 2.9. Animals

Male *db/db* mice (6 weeks old) were obtained from GemPharmatech Co., Ltd. (Nanjing, China; Animal License No. T002407DB) and housed in a specific-pathogen-free facility under controlled conditions (12 h light/dark cycle, 20–25 °C, 40–50% humidity). After 1-week acclimation, mice were used for experiments. MAFLD model establishment: Thirty 6-week-old db/db mice were randomized into three dietary groups: VD-deficient: 25 IU VD_3_/3600 kcal; VD-normal: 1000 IU VD_3_/3600 kcal; VD-supplemented: 5000 IU VD_3_/3600 kcal. Mice were fed modified standard chow (Trophic Animal Feed High-tech Co., Ltd., Nantong, China) for 12 weeks. After overnight fasting, mice were anesthetized, and serum/liver tissues were collected for analysis. All procedures followed the NIH Guide for the Care and Use of Laboratory Animals and were approved by the Animal Ethics Committee of Gansu University of Traditional Chinese Medicine.

### 2.10. Histological Analysis

After 12 weeks of treatment, db/db mice were anesthetized, and liver tissues were collected and fixed in 4% paraformaldehyde (PFA). The fixed tissues were paraffin-embedded and sectioned into 5 μm thick slices for hematoxylin–eosin (H&E) and immunohistochemistry (IHC) staining. IHC staining was performed using antibodies against LC3B (1:500, GTX127375, GeneTex), P62 (1:200, GTX100685, GeneTex), NLRP3 (1:100, TD7348, Abmart), Caspase-1 (1:100, GTX101322, GeneTex), and GSDMD (1:500, ab219800, Abcam). Staining reagents and technical support were provided by the Pathology Department of Gansu Province Hospital. Images were acquired using an Olympus microscope.

### 2.11. Serum Biochemical Analysis

Serum biochemical parameters, including total cholesterol (TC), triglycerides (TG), fasting blood glucose (FBG), high-density lipoprotein cholesterol (HDL-C), low-density lipoprotein cholesterol (LDL-C), alanine aminotransferase (ALT), and aspartate aminotransferase (AST), were analyzed using an automated biochemical analyzer (Chemray 240/420/800, Nanjing, China). Serum concentrations of interleukin-1β (IL-1β), interleukin-18 (IL-18), and 25-hydroxyvitamin D3 [25(OH)D3] were quantified by sandwich ELISA kits (Elabscience, Wuhan, China) following the manufacturer’s protocols.

### 2.12. Statistical Analysis

Data are expressed as mean ± SD from ≥3 independent experiments. Statistical analyses were performed using SPSS 20.0 and GraphPad Prism 6. One-way ANOVA with Bonferroni post hoc test was applied for multi-group comparisons. Statistical significance was defined as *p* < 0.05.

## 3. Results

### 3.1. 1,25(OH)_2_D_3_ Protects Against HG-Induced HLSEC Capillarization

**Figure 1:**
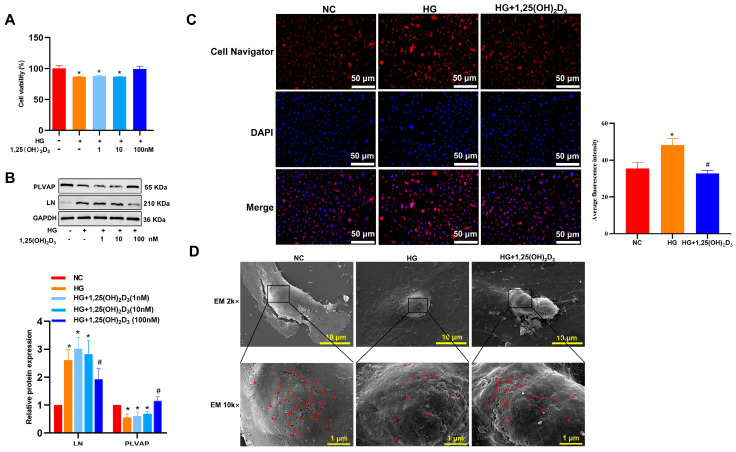
Vitamin D improves hepatic sinusoidal capillarization induced by high glucose. (**A**) Effects of vitamin D on cell viability in HLSECs. (**B**) The expression of LN and PLVAP proteins following exposure to HG and VD. The protein fraction was analyzed by Western blotting. (**C**) Effects of vitamin D on the HG-induced accumulation of lipids. (**D**) Ultrastructural features of HG-treated HLSECs by scanning electron microscopy. The red triangular mark represents the fenestra structures. * *p* < 0.05 compared with the control group; ^#^ *p* < 0.05 vs. HG-treated group. Abbreviations: NC, normal control; HG, high glucose; LN, laminin; PLVAP, plasmalemma vesicle-associated protein.

HLSECs were exposed to different D-glucose concentrations (5.5, 11, 16.5, 25, 30 mM) for 12, 24, or 48 h. Western immunoblotting showed that high-glucose (HG, 25 and 30 mM) treatment for 24 and 48 h significantly increased LN expression and decreased PLVAP expression compared to the low-glucose (5.5 mM) control (Appendix A). When HLSECs were treated with a fixed 25 mM HG and different 1,25(OH)_2_D_3_ doses (1, 10, 100 nM) for 24 h, HG reduced cell viability compared to the control, but 1,25(OH)_2_D_3_ (100 nM) reversed this effect (Figure 1A). Western immunoblotting further demonstrated that 1,25(OH)_2_D_3_ supplementation rescued the HG-induced changes in LN and PLVAP expression (Figure 1B). Cell Navigator fluorescence analysis indicated that HG promoted more lipid accumulation in HLSECs than in control cells, while 1,25(OH)_2_D_3_ prevented this (Figure 1C). Scanning electron microscopy (SEM) of fenestra structures confirmed that HG decreased fenestra numbers compared to the control, and 1,25(OH)_2_D_3_ reversed these changes (Figure 1D). To conclude, HG can induce hepatic sinusoid capillarization, which can be attenuated by 1,25(OH)_2_D_3_ supplementation.

### 3.2. 1,25(OH)_2_D_3_ Inhibits HG-Induced Pyroptosis in HLSECs to Protect Against Hepatic Sinusoidal Capillarization

**Figure 2:**
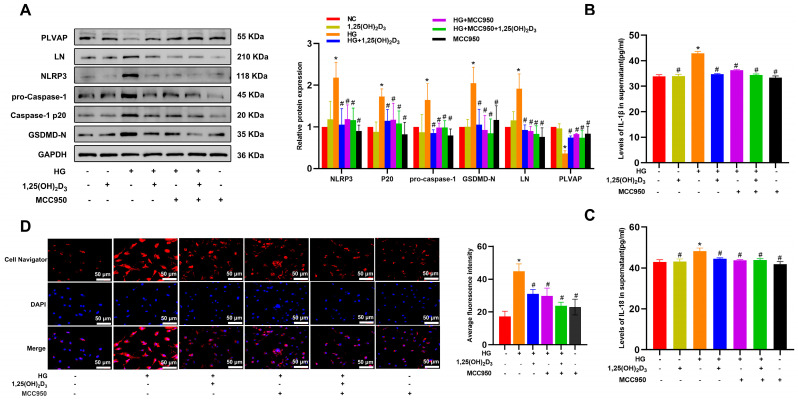
Inhibitory effect of vitamin D on HG-induced pyroptosis and hepatic sinusoidal capillarization of HLSECs. (**A**) Effects of MCC950 and vitamin D on pyroptosis-related markers, hepatic sinus structural protein (PLVAP), and basement membrane protein (LN) in HG-treated HLSECs. (**B**,**C**) The IL-1β and IL-18 content in HLSEC culture supernatants were determined by ELISA. (**D**) Lipid accumulation in HLSECs was assessed by Cell Navigator fluorescence. Scale bar = 50 μm. * *p* < 0.05 compared with the control group; ^#^ *p* < 0.05 vs. HG-treated group. Abbreviations: HG, high glucose; LN, laminin; PLVAP, plasmalemma vesicle-associated protein; NLRP3, NLR family pyrin domain containing 3; GSDMD, gasdermin D; IL, interleukin; Caspase, cysteinyl aspartate specific proteinase.

Western immunoblotting showed that HG treatment upregulated pyroptosis-related proteins (NLRP3, Caspase-1 p20, GSDMD-N) in HLSECs, and 1,25(OH)_2_D_3_ suppressed these changes (Appendix B). To clarify the link between NLRP3 inflammasome activity and pyroptosis under HG and 1,25(OH)_2_D_3_ treatment, cells were treated with 1 μM of the NLRP3 inhibitor MCC950 for 24 h in 25 mM HG, with or without 1,25(OH)_2_D_3_. HG increased LN, NLRP3, Caspase-1 p20, and GSDMD-N levels and decreased PLVAP levels, which were reversed by 1,25(OH)_2_D_3_ or MCC950 (Figure 2A). ELISA revealed that HG elevated IL-1β and IL-18 production compared to controls, and both MCC950 and 1,25(OH)_2_D_3_ mitigated these inflammatory responses (Figure 2B,C). 1,25(OH)_2_D_3_ and MCC950 both inhibited HG-induced lipid accumulation in HLSECs (Figure 2D). Collectively, these data suggest 1,25(OH)_2_D_3_ attenuates HG-induced pyroptotic cell death, alleviating hepatic sinusoid capillarization.

### 3.3. 1,25(OH)_2_D_3_ Promotes Autophagic Activity in HG-Treated HLSECs to Alleviate Hepatic Sinusoidal Capillarization

**Figure 3:**
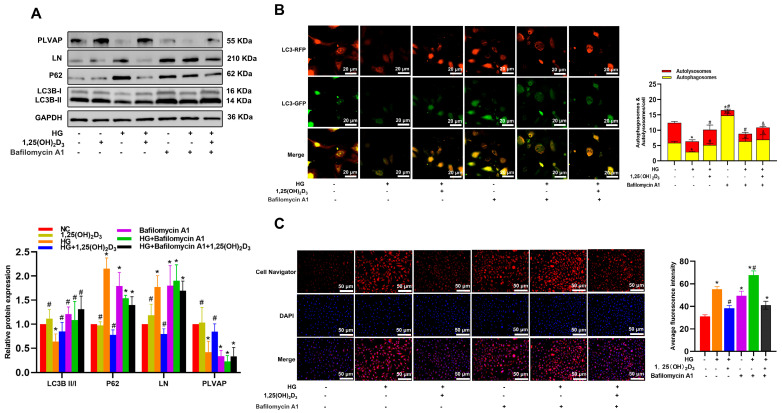
VD treatment induces autophagy and restores autophagic flux to alleviate hepatic sinusoidal capillarization. (**A**) HLSECs were cultured in the presence of HG with or without VD and treated with Baf-A1 for 24 h. The protein levels of autophagy-related markers, hepatic sinus structural protein (PLVAP), and basement membrane protein (LN) were determined by Western blotting. (**B**) Confocal microscopy observation of mRFP-GFP-LC3 adenovirus transfected HLSECs treated as indicated (magnification: ×40; scale bars = 20 μm). (**C**) Lipid accumulation in HLSECs was assessed by Cell Navigator fluorescence. Scale bar = 50 μm. * *p* < 0.05 compared with the control group; ^#^ *p* < 0.05 vs. HG-treated group; ^&^ *p* < 0.05 compared with the HG + Bafilomycin A1. Abbreviations: HG, high glucose; LN, laminin; PLVAP, plasmalemma vesicle-associated protein; LC3, microtubule-associated proteins light chain 3.

After 24 h of treatment with various HG concentrations, Western immunoblotting showed that LC3-II levels in HLSECs decreased and P62 levels increased with rising glucose concentrations. 1,25(OH)_2_D_3_ reversed these changes (Appendix C). To confirm 1,25(OH)_2_D_3_’s ability to induce autophagy in HG-treated HLSECs and protect against hepatic sinusoidal capillarization, cells were treated with the autophagy inhibitor Bafilomycin A1 (Baf-A1, 10 nM), 25 mM HG, and/or 1,25(OH)_2_D_3_ for 24 h. Baf-A1, which blocks autophagosome–lysosome fusion, upregulated P62 and LC3B-II in these cells (Figure 3A). Transfection of HLSECs with the mRFP-GFP-LC3 adenoviral vector further demonstrated that HG suppressed autophagy, while 1,25(OH)_2_D_3_ restored it (Figure 3B). Western immunoblotting revealed that HG significantly increased LN expression and decreased PLVAP levels compared to controls. 1,25(OH)_2_D_3_ reversed these changes, and Baf—A1 enhanced LN and reduced PLVAP levels in HLSECs (Figure 3A). 1,25(OH)_2_D_3_ inhibited HG-induced lipid accumulation, while Baf-A1 exacerbated it (Figure 3C). Collectively, these results indicate that 1,25(OH)_2_D_3_ can induce autophagy, thus alleviating hepatic sinusoidal capillarization.

### 3.4. 1,25(OH)_2_D_3_ Induces Autophagy to Protect Against HLSEC Pyroptosis

**Figure 4:**
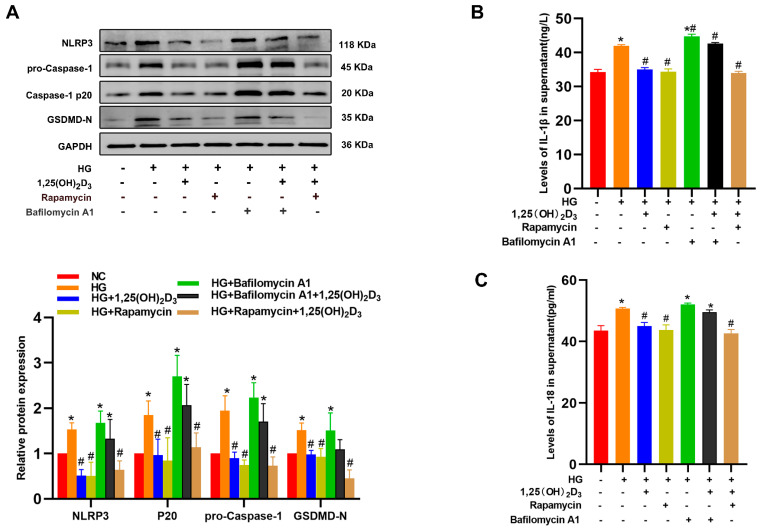
VD protects HLSECs from HG-induced pyroptotic cell death by promoting autophagy. HLSECs were cultured in the presence of HG with or without VD and subsequently treated with rapamycin, Baf-A1 for 24 h. (**A**) The protein levels of pyroptosis-related markers were determined by Western blotting. (**B**,**C**) The IL-1β and IL-18 content in HLSEC culture supernatants was determined by ELISA. * *p* < 0.05 compared with the control group; ^#^ *p* < 0.05 vs. HG-treated group. Abbreviations: HG, high glucose; NLRP3, NLR family pyrin domain containing 3; IL, interleukin; Caspase, cysteinyl aspartate specific proteinase; IL, interleukin.

To better clarify autophagy’s role in 1,25(OH)_2_D_3_-mediated pyroptosis inhibition in HG-treated HLSECs, these cells were treated with Baf-A1 (10 nM), autophagy agonist rapamycin (20 nM), and/or 1,25(OH)_2_D_3_. In the presence of Baf-A1, 1,25(OH)_2_D_3_ lost its ability to inhibit NLRP3, Caspase-1, GSDMD-N, IL-1β, and IL-18. In contrast, 1,25(OH)_2_D_3_ treatment effects resembled those of rapamycin (Figure 4A–C). Collectively, these results indicate that 1,25(OH)_2_D_3_ protects HLSECs from pyroptotic death by inducing autophagy.

### 3.5. 1,25(OH)_2_D_3_ May Control Autophagy Through the AMPK/mTOR Pathway to Prevent Pyroptosis and Hepatic Sinusoidal Capillarization

**Figure 5:**
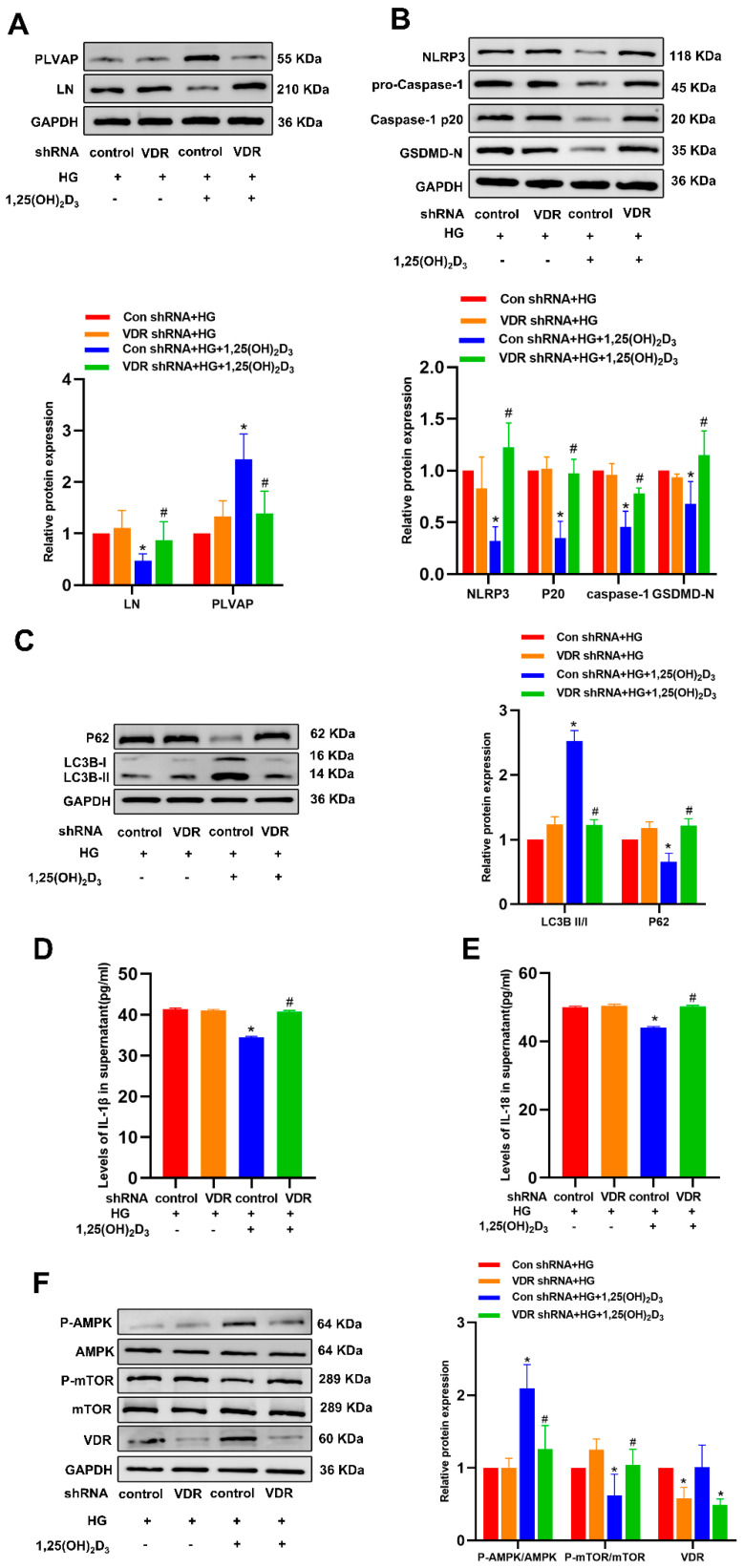
VD regulates HLSEC autophagy, pyroptosis, and AMPK/mTOR signals via VDR. At post-transfection with VDR-specific or control shRNA constructs, HLSECs were treated with HG (25 mM) with or without 1,25(OH)_2_D_3_ (100 nM) for 24 h. (**A**–**C**,**F**) Western immunoblotting was then used to assess LN, PLVAP, p62, LC3BII/I, NLRP3, Caspase-1 p20, GSDMD-N, VDR, p-AMPK/AMPK, and p-mTOR/mTOR levels. (**D**,**E**) The IL-1β and IL-18 content in HLSEC culture supernatants was determined by ELISA. * *p* < 0.05 vs. corresponding Con shRNA + HG group. ^#^ *p* < 0.05 vs. corresponding Con shRNA + HG +1,25(OH)_2_D_3_ group. Abbreviations: HG, high glucose; shRNA, short hairpin RNA; VDR, vitamin D receptor; LN, laminin; PLVAP, plasmalemma vesicle-associated protein; NLRP3, NLR family pyrin domain containing 3; IL, interleukin; Caspase, cysteinyl aspartate specific proteinase; LC3, microtubule-associated proteins light chain 3; P-AMPK, phosphorylated AMPK; P-mTOR, phosphorylated mTOR.

Since the signaling of 1,25(OH)_2_D_3_ is mediated through the vitamin D receptor (VDR), the short hairpin RNA (shRNA) technique was subsequently employed to knock down VDR. This approach provides a direct means to evaluate the importance of VDR signaling in the context of this experiment (Appendix D). The knockdown of VDR significantly eliminated the effects of 1,25(OH)_2_D_3_ on the expression of LN, PLVAP, P62, LC3BII/I, NLRP3, Caspase-1 p20, GSDMD-N, IL-1β, and IL-18, highlighting the crucial role of VDR in this situation (Figure 5A–E). It is worth noting that rapamycin induces autophagy by inhibiting the expression of the mammalian target of rapamycin (mTOR). Therefore, we hypothesized that similar to rapamycin, 1,25(OH)_2_D_3_ triggers autophagy in HLSECs through the regulation of the AMP-activated protein kinase/mammalian target of rapamycin (AMPK/mTOR) signaling pathway. Western blot analysis after HG treatment showed that the expression of phosphorylated AMPK decreased while the expression of phosphorylated mTOR increased. However, 1,25(OH)_2_D_3_ effectively counteracted the effects of high glucose (Figure 5F).

**Figure 6:**
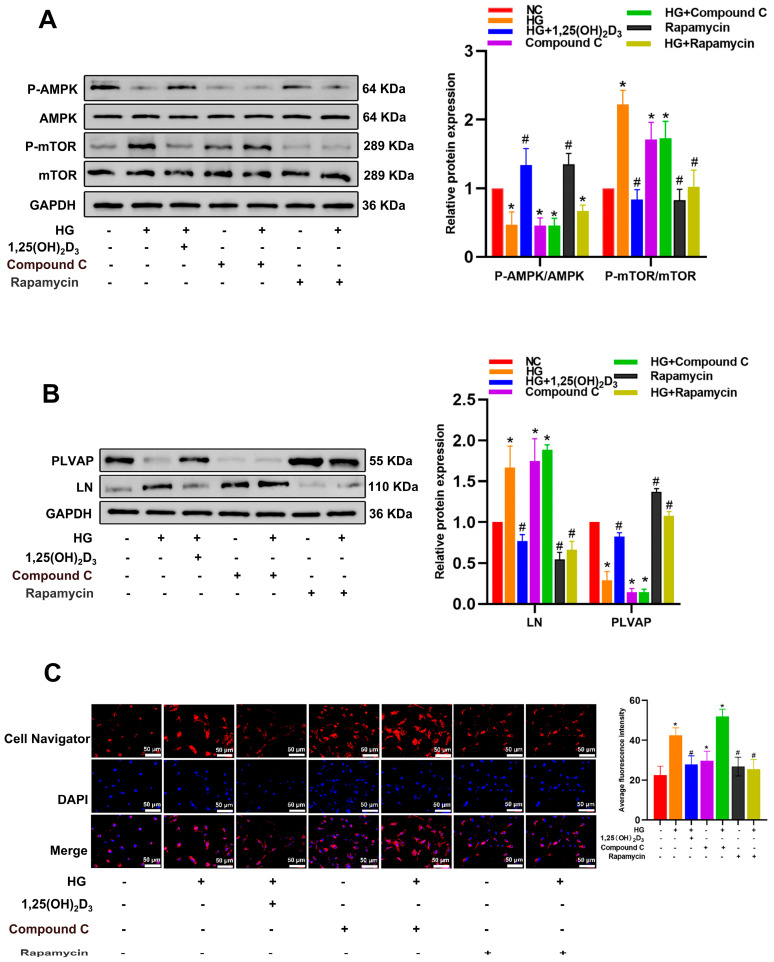
The importance of AMPK/mTOR signaling downstream of VD signaling in this model of HG-induced hepatic sinusoidal capillarization. (**A**) Western blotting was used to detect the protein level of p-AMPK and AMPK and p-mTOR and mTOR. (**B**) Detection of LN and PLVAP levels after Compound C and rapamycin treatments. (**C**) Lipid accumulation in HLSECs was assessed by Cell Navigator fluorescence. Scale bar = 50 μm. * *p* < 0.05 compared with the control group; ^#^ *p* < 0.05 vs. HG-treated group. Abbreviations: HG, high glucose; LN, laminin; PLVAP, plasmalemma vesicle-associated protein; P-AMPK, phosphorylated AMPK; P-mTOR, phosphorylated mTOR.

**Figure 7:**
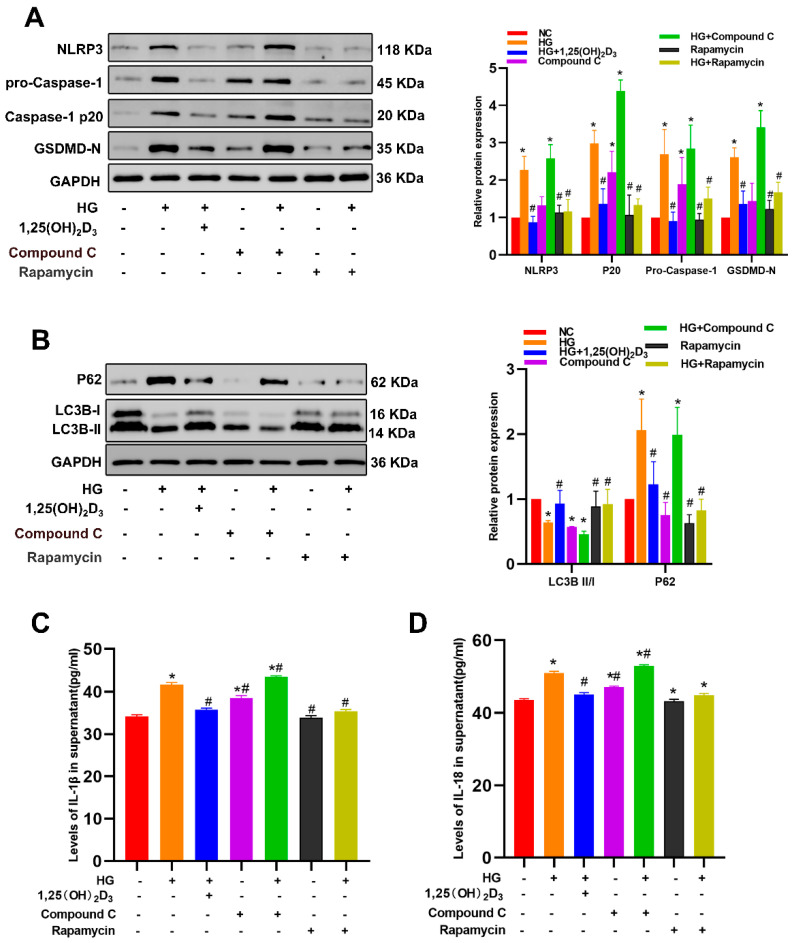
(**A**,**B**) The protein levels of autophagy and pyroptosis-related markers were determined by Western blotting. (**C**,**D**) The IL-1β and IL-18 content in HLSEC culture supernatants was determined by ELISA. Abbreviations: NLRP3, NLR family pyrin domain containing 3; IL, interleukin; Caspase, cysteinyl aspartate specific proteinase; LC3, microtubule-associated proteins light chain 3. * *p* < 0.05 compared with the control group; *^#^ p* < 0.05 vs. HG-treated group.

To determine the significance of AMPK/mTOR signaling downstream of 1,25(OH)_2_D_3_ in HG-induced hepatic sinusoidal capillarization, HG-treated HLSECs were treated with rapamycin or Compound C (AMPK inhibitor, 10 μM) for 24 h. Compound C decreased p-AMPK-AMPK but increased p-mTOR-mTOR (Figure 6A). In HLSECs treated with HG, Compound C significantly reduced PLVAP protein levels and increased LN expression and intracellular lipid accumulation. Compared with the HG treatment group, rapamycin significantly increased PLVAP expression, significantly decreased LN protein levels, and reduced intracellular lipid accumulation (Figure 6B,C). Compound C significantly decreased LC3B protein levels while increasing the expression of P62, NLRP3, Caspase-1 p20, GSDMD-N, IL-1β, and IL-18. Compared with the HG treatment group, rapamycin significantly increased LC3B expression and, conversely, significantly decreased the protein levels of P62, NLRP3, Caspase-1 p20, GSDMD-N, IL-1β, and IL-18 (Figure 7). These results suggest that HG inhibits autophagy by suppressing the AMPK-mTOR signaling pathway, inducing pyroptosis and hepatic sinusoidal endothelial cell injury. 1,25(OH)_2_D₃ can activate AMPK and inhibit mTOR phosphorylation. These findings indicate that HG suppresses autophagy and induces pyroptosis and HLSEC injury via inhibiting AMPK-mTOR signaling. 1,25(OH)_2_D_3_ can activate AMPK and inhibit mTOR phosphorylation.

### 3.6. Vitamin D Supplementation Suppresses the Development of MAFLD in db/db Mice

To investigate the regulatory effects of vitamin D on lipid metabolic activity and the progression of MAFLD, *db/db* mice were administered standard rodent chow containing varying concentrations of vitamin D for a 12-week period. Serum 25(OH)D3, a reliable biomarker of vitamin D status, demonstrated that a vitamin D-deficient (VD def) diet-induced pronounced 25(OH)D3 deficiency, whereas a vitamin D-supplemented (VD sup) diet elevated 25(OH)D3 levels. In comparison with the normal control and VD sup groups, VD def mice exhibited significantly elevated levels of triglyceride (TG), total cholesterol (TC), aspartate aminotransferase (AST), and alanine aminotransferase (ALT) (*p* < 0.01) yet significantly reduced levels of high-density lipoprotein cholesterol (HDL-C) (*p* < 0.01). Fasting blood glucose (FBS) and low-density lipoprotein cholesterol (LDL-C) levels did not differ significantly among the three groups (*p* > 0.01). These findings suggest that vitamin D supplementation can ameliorate the serum lipid profiles in this murine MAFLD model (Table 1).

**Figure 8:**
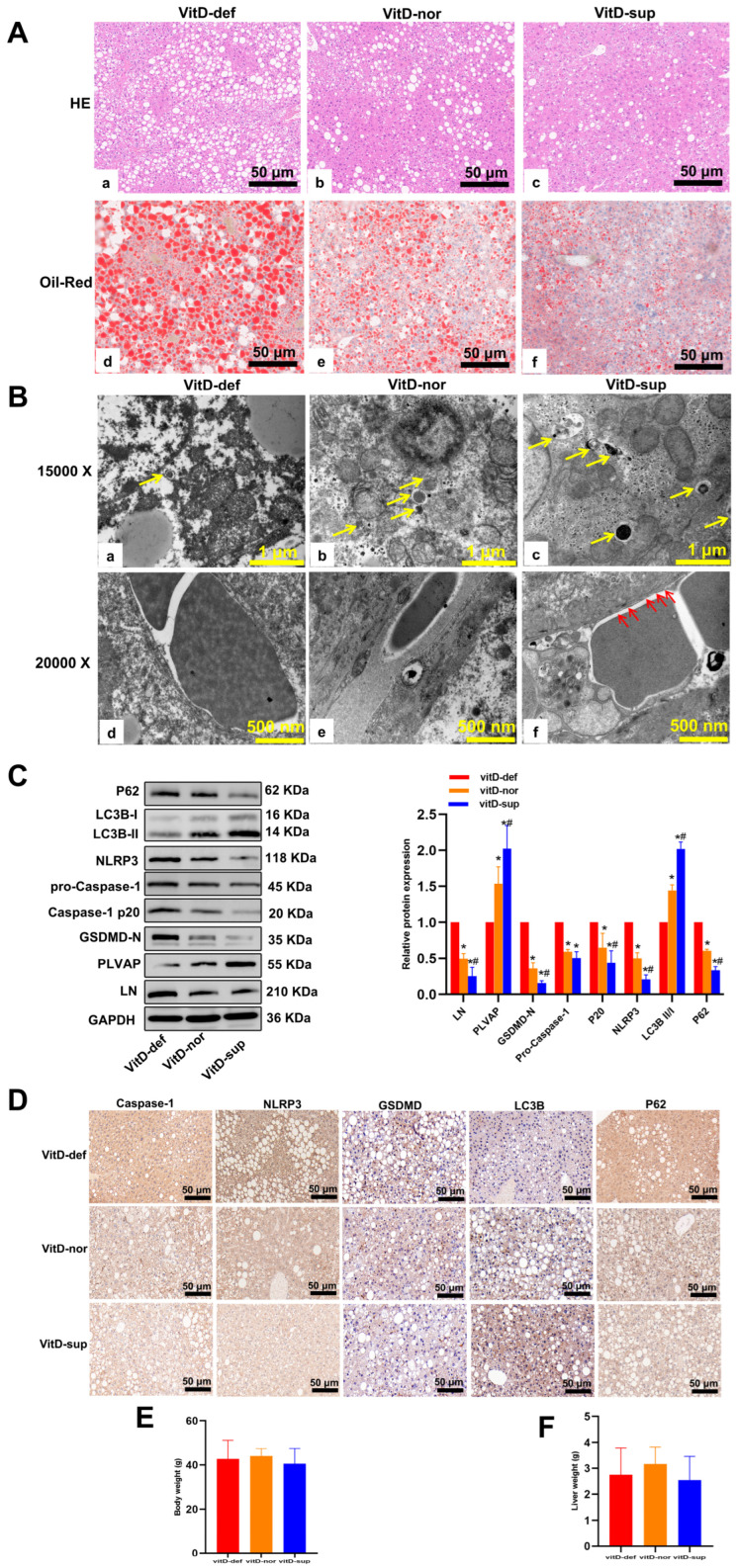
Vitamin D suppresses the development of diabetic non-alcoholic fatty liver disease in db/db mice. Db/db mice were fed a standard chow containing various vitamin D doses (vitD def, vitD nor, and vitD Sup) for 12 weeks. (**A**) HE and Oil Red O staining of liver sections after treatment with VD. Scale bar = 50 μm. (**B**) Ultrastructural features of VD-treated mouse livers by transmission electron microscopy. Yellow arrows indicate autophagosomes (scale bar = 1 μm). Red arrows indicate fenestra (scale bar = 500 nm). (**C**) The protein levels of LN, PLVAP, autophagy- and pyroptosis-related markers were determined by Western blotting. (**D**) Representative immunohistochemical staining of autophagy- and pyroptosis-related protein in three groups (40×). (**E**,**F**) The body weight and liver weight. Results are means ± SD, * *p* < 0.05 vs. Vit D def group, ^#^ *p* < 0.05 vs. Vit D nor group. Abbreviations: VitD-def, Vitamin D deficient; VitD-nor, Vitamin D normal; VitD-sup, Vitamin D supplementation.

To directly evaluate vitamin D’s effect on MAFLD development, H&E and Oil Red O staining were performed on mouse liver tissue. VD-deficient mice had significantly more hepatic lipid build-up and steatosis than VD-supplemented and normal control groups (Figure 8A). TEM showed that VD-deficient LSECs had fewer autophagosomes, thicker basement membranes, and lost fenestra structures compared to VD-supplemented ones (Figure 8B). VD-deficient mice had higher LN and lower PLVAP expression. Western blotting and IHC confirmed higher LC3-II in VD-supplemented mice, which was the opposite for P62. VD-deficient mice had increased NLRP3, Caspase-1, and GSDMD-N expression (Figure 8C,D) and higher serum IL-1β and IL-18 levels (*p* < 0.01, Table 1). Body and liver weights were similar among the three groups (Figure 8E,F).

## 4. Discussion

Here, the effects of 1,25(OH)_2_D_3_ on HG-exposed LSECs were analyzed at length. The present study demonstrated that HG downregulated the level of autophagy and triggered NLRP3 inflammasome activation, leading to pyroptotic cell death, and these effects could be reversed by 1,25(OH)_2_D_3_. In MAFLD model mice, VD supplementation was confirmed to elicit autophagic activation while suppressing pyroptotic cell death, ultimately protecting against hepatic damage. Restoring autophagic activity was also found to inhibit NLRP3 inflammasome activity and pyroptotic cell death. The data gathered in this study suggest that 1,25(OH)_2_D_3_ alleviated the pyroptosis of HLSECs exposed to HG by promoting cell autophagy via the AMPK–mTOR axis.

MAFLD development involves a multifactorial process mediated by tightly regulated mechanisms, where endothelial dysfunction and hepatic sinusoidal remodeling constitute critical pathogenic components. Emerging evidence indicates that liver sinusoidal endothelial cell (LSEC) capillarization and concomitant inflammatory responses synergistically drive disease progression [25,26]. Thus, elucidating the molecular mechanisms underlying LSEC capillarization and inflammation represents a crucial strategy for developing therapeutic interventions against MAFLD advancement.

NLRP3 inflammasome activation drives pro-inflammatory pyroptosis and contributes to MAFLD pathogenesis [27]. While NLRP3 activation is enhanced in TNF-α/BZATP-stimulated LSECs [28], no reports to date have explored whether NLRP3 inflammasome-related pyroptosis plays a role in hepatic sinusoid capillarization. Here, HG treatment was used to induce HLSEC capillarization, with treated HLSECs exhibiting pyroptotic features, including elevated NLRP3, Caspase-1 p20, GSDMD-N terminal, and pro-inflammatory cytokines (IL-1β, IL-18), alongside decreased cell viability. MCC950 attenuated pyroptosis and lipid deposition while suppressing laminin expression and enhancing PLVAP levels in HG-treated HLSECs. These findings implicate LSEC pyroptosis as a regulatory mechanism in sinusoidal capillarization.

Emerging evidence positions vitamin D as a therapeutic modulator in metabolic disorders, particularly through VDR-mediated anti-inflammatory actions in hepatic and extrahepatic tissues [29]. Clinically, MAFLD progression correlates with vitamin D deficiency, as evidenced by inverse associations between serum vitamin D levels and hepatic fibrosis scores [30]. Mechanistically, vitamin D demonstrates dual protective capacities: (1) Attenuating NLRP3 inflammasome activation to suppress pyroptosis in metabolic-stressed cells, as shown in LPS-challenged hepatocytes [24] and hyperglycemic pancreatic β-cells [31]. (2) Preserving vascular homeostasis through endothelial protection.

Our experimental data reveal that 1,25(OH)_2_D_3_ mitigates HG-induced HLSEC capillarization by concurrently inhibiting NLRP3-dependent pyroptosis (evidenced by reduced NLRP3, Caspase-1 p20, GSDMD-N, IL-1β, and IL-18) and reversing capillarization markers (downregulated LN, upregulated PLVAP). These effects were VDR-dependent, as VDR knockdown abolished the protective phenotype. In vivo validation using db/db mice confirmed vitamin D’s capacity to ameliorate hepatic steatosis, suppress pyroptotic signaling, and maintain sinusoidal architecture. Collectively, these findings establish that vitamin D can prevent capillarization of LSECs through its effect on NLRP3-associated pyroptotic cell death.

Autophagic flux maintains cellular homeostasis and serves as a crucial regulator of hepatic lipid metabolism. Autophagy impairment drives hepatic steatosis and MAFLD pathogenesis by disrupting lipid processing [32]. This mechanistic link positions autophagy enhancement as a potential therapeutic strategy against MAFLD progression. Supporting this concept, metformin demonstrates dual benefits in primary hepatocytes and ob/ob mice by inducing autophagy to ameliorate hepatic inflammation, lipid accumulation, and glucose dysregulation [33]. Lim et al. demonstrated that VD3 was able to increase the hepatic activity of LC3B-II activity while attenuating hepatic steatosis in T2DM mice fed a high-fat diet [34]. We found that in T2DM-like db/db mice, VD3 deficiency was associated with hepatocyte lipid accumulation, hepatic steatosis, reduced LC3B, and increased expression of P62, together with decreases in autophagosome formation. However, supplementation with vitamin D3 reversed these effects. In vitro, we demonstrated that 1,25(OH)_2_D_3_ prevented sinusoidal capillarization by inducing autophagy in HLSECs. Knocking down VDR eliminated the ability of 1,25(OH)_2_D_3_ to induce autophagy, supporting its ability to directly induce autophagic activity when applied in the context of MAFLD. The effects of 1,25(OH)_2_D_3_ have been reported to vary among model systems. In a model of diabetic heart injury, for instance, vitamin D reportedly attenuated diabetes-related cardiac damage and autophagy through VDR-mediated signaling and the consequent inhibition of FoxO1 translocation to the nucleus [35]. In BT474 breast cancer cells, vitamin D can also derepress autophagy, with these cells expressing low levels of VDR [36]. These varied results may be related to cell or tissue-specific patterns of VDR expression or other aspects of the downstream targets of VDR signaling. Rapamycin-induced autophagy also significantly reduced lipid accumulation in HLSEC and, at the same time, prevented pyroptosis and capillarization. 1,25(OH)_2_D_3_ exerted beneficial effects in line with those induced by rapamycin. Inhibiting autophagy, however, disrupted the beneficial effects of 1,25(OH)_2_D_3_ in this model system, suggesting that 1,25(OH)_2_D_3_ can inhibit HG-induced HLSEC capillarization at least in part via enhancing autophagy, thereby preventing the accumulation of lipids and inhibiting pyroptosis driven by NLRP3 activity.

To determine whether AMPK/mTOR signaling mediates 1,25(OH)_2_D_3_-induced autophagy in HG-exposed HLSECs, we conducted mechanistic studies based on established AMPK/mTOR-autophagy axis evidence [14,37,38]. As a sensor of cellular energy status, AMPK positively regulates autophagy and can modify energetic metabolism through reductions in the phosphorylation of mTOR, which functions as a kinase upstream of autophagy-related genes such that its activity can suppress autophagy, whereas the activation of AMPK negatively regulates mTOR, ultimately favoring the onset of autophagy [39]. In this study, HG-treated HLSECs exhibited increased mTOR phosphorylation together with a reduction in AMPK phosphorylation, while 1,25(OH)_2_D_3_ treatment was sufficient to reverse these trends. The knockdown of VDR was also sufficient to ablate the effects of 1,25(OH)_2_D_3_. Compound C (AMPK inhibitor) significantly reduced autophagy of HLSECs and increased pyroptosis, while rapamycin has the opposite effect to compound C. Rapamycin, which acts through specific inhibition of mTOR phosphorylation in the AMPK/mTOR pathway, is recognized as a drug that promotes autophagy, whereas 1,25(OH)_2_D_3_ exerted beneficial effects in line with those induced by rapamycin. These results suggest that the induction of autophagy by 1,25(OH)_2_D_3_ may be mediated by activating the AMPK/mTOR signaling pathway.

In the animal study, the overall liver tissue analysis method was employed in this experiment. Although the regulatory effects of vitamin D intervention on the inflammasome and autophagy pathway were observed, due to the lack of specific analysis of LSECs, the impact of vitamin D on hepatocytes cannot be excluded. However, the pathological results demonstrated that vitamin D intervention improved the fenestration structure of LSECs, and the direct effect of vitamin D on LSECs was further verified through the in vitro culture of LSECs.

## 5. Conclusions

Taken together, our results demonstrated that vitamin D—VDR can activate autophagy and inhibit pyroptosis in liver sinusoidal endothelial cells to improve liver sinusoidal capillarization. In the livers of mice with MAFLD, autophagy is suppressed while pyroptosis is enhanced. Supplementation with vitamin D can trigger autophagic activation, inhibit pyroptosis, and protect the liver from damage. The AMPK/mTOR signaling pathway may be involved in the effects of vitamin D on autophagic activation and pyroptosis inhibition. Therefore, our study may provide a theoretical basis for the application of vitamin D in the treatment of type 2 diabetes mellitus complicated with MAFLD.

## Figures and Tables

**Table 1:** VD supplementation to improve the serum lipid profiles in this murine MAFLD model.

Parameter	vitD-def	vitD-nor	vitD-sup	*p* Value
25(OH)D_3_ (ng/mL)	19.12 ± 0.25	33.02 ± 1.19 *	59.74 ± 2.11 *^#^	<0.01
FBG (mmol/L)	31.17 ± 2.86	32.39 ± 2.65	33.43 ± 2.94	>0.01
TG (mmol/L)	2.28 ± 0.10	1.74 ± 0.08 *	1.17 ± 0.05 *^#^	<0.01
TC (mmol/L)	7.59 ± 0.47	6.07 ± 0.27 *	4.44 ± 0.39 *^#^	<0.01
HDL-C (mmol/L)	1.18 ± 0.13	2.30 ± 0.13 *	2.43 ± 0.17 *^#^	<0.01
LDL-C (mmol/L)	0.41 ± 0.04	0.50 ± 0.07	0.60 ± 0.12	>0.01
ALT (U/L)	236.10 ± 8.97	180.70 ± 10.05 *	141.8 ± 10.89 *^#^	<0.01
AST (U/L)	418.80 ± 16.64	340.40 ± 10.42 *	281.60 ± 11.38 *^#^	<0.01
IL-1β (pg/mL)	14.41 ± 0.60	6.42 ± 0.51 *	3.81 ± 0.23 *^#^	<0.01
IL-18 (pg/mL)	1149 ± 57.54	787.0 ± 28.46 *	610.3 ± 37.49 *^#^	<0.01

Serum lipid (TC, TG, HDL-C, LDL-C), ALT, AST, GLU, 25(OH)D_3_, IL-1β, and IL-18 levels were then assessed in three groups (n = 10) via automatic biochemical analyzer and ELISA, respectively. Data are means ± SD. * *p* < 0.01 vs. Vit D def group, ^#^ *p* < 0.01 vs. Vit D nor group. Abbreviations: FBG, fasting blood glucose; TG, triglyceride; TC, total cholesterol; HDL-C, high-density lipoprotein cholesterol; LDL-C, low-density lipoprotein cholesterol; ALT, alanine transaminase; AST, aspartate aminotransferase; IL, interleukin.

**Figure A1:**
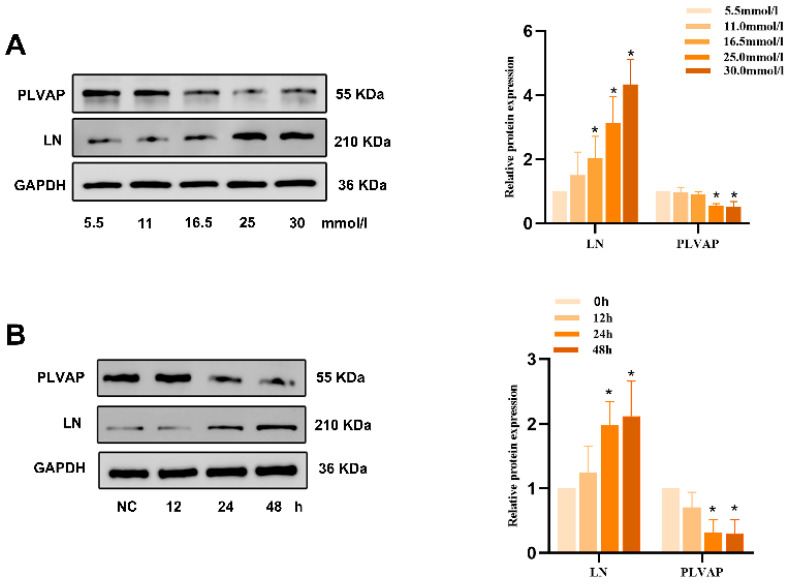
The effects of different concentrations of glucose on the expression of LN and PLVAP in HLSECs at different time points. (**A**,**B**) The expression of the hepatic sinus structural protein (PLVAP) and basement membrane protein (LN) following exposure to HG. The protein fraction was analyzed by Western blotting. * *p* < 0.05 compared with the control group. Abbreviations: NC, normal control; HG, high glucose; LN, laminin; PLVAP, plasmalemma vesicle-associated protein.

**Figure A2:**
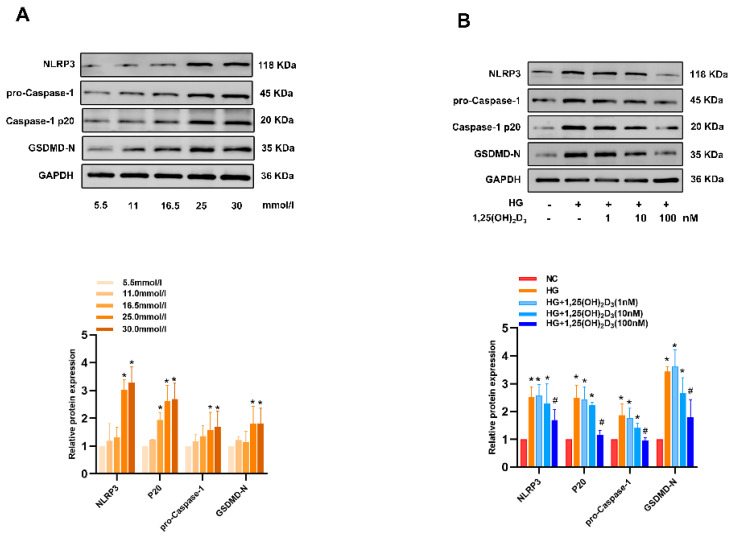
The effects of different concentrations of 1,25(OH)_2_D_3_ on pyroptosis of HLSECs induced by HG. (**A**) The expression of pyroptosis-related proteins following exposure to HG. (**B**) HLSECs were treated with 1,25(OH)_2_D_3_ (1, 10, or 100 nM) and with HG (25 mM) for 24 h. Western blotting was then used to detect the pyroptosis-related proteins. * *p* < 0.05 compared with the control group; ^#^ *p* < 0.05 vs. HG-treated group. Abbreviations: HG, high glucose; NLRP3, NLR family pyrin domain containing 3; Caspase, cysteinyl aspartate specific proteinase.

**Figure A3:**
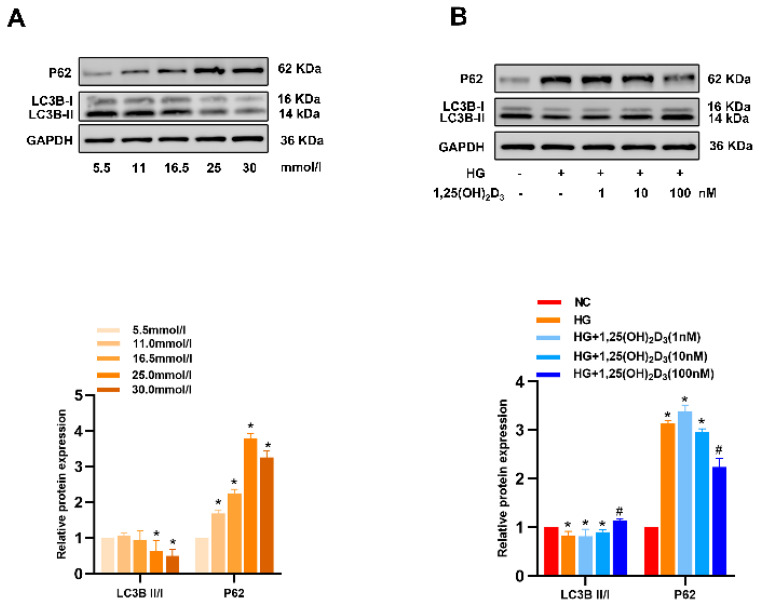
The effects of different concentrations of 1,25(OH)_2_D_3_ on autophagy of HLSECs induced by HG. (**A**) The expression of autophagy-related proteins following exposure to HG. (**B**) HLSECs were incubated with VD (1, 10, 100 nM) in the presence of HG (25 mM) for 24 h. Protein levels of LC3B and P62 were determined by Western blotting. * *p* < 0.05 compared with the control group; ^#^ *p* < 0.05 vs. HG-treated group. Abbreviations: NC, normal control; HG, high glucose; LC3, microtubule-associated proteins light chain 3.

**Figure A4:**
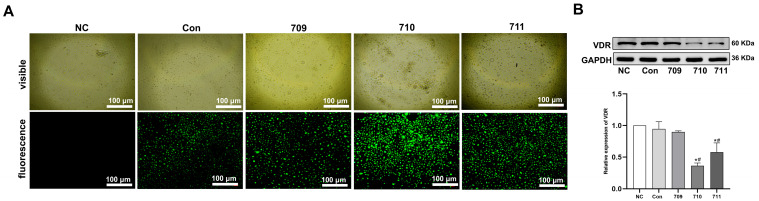
ShRNA transfection reduces the expression of VDR in hepatic sinusoidal endothelial cells. (**A**,**B**) HLSECs were infected with three expression vectors targeting VDR and a negative control vector for 72 h, respectively. The knockdown efficiency of VDR was confirmed by Western blotting and immunofluorescence. * *p* < 0.05 vs. control group. ^#^ *p* < 0.05 vs. negative control group.

## Data Availability

The datasets analyzed during the current study are available from the corresponding author upon reasonable request.

## Abbreviations

The following abbreviations are used in this manuscript:

MAFLD, metabolic dysfunction-associated fatty liver disease; T2DM, type 2 diabetes mellitus; LSECs, liver sinusoidal endothelial cells; VD, vitamin D; HLSECs, human LSECs; FBG, fasting blood glucose; Baf-A1, Bafilomycin A1;NC, normal control; HG, high glucose; LN, laminin; PLVAP, plasmalemma vesicle-associated protein; NLRP3, NLR family pyrin domain containing 3; IL, interleukin; Caspase, cysteinyl aspartate specific proteinase; LC3, microtubule-associated proteins light chain 3; shRNA, short hairpin RNA; VDR, vitamin D receptor; AMPK, AMP-activated protein kinase; P-AMPK, phosphorylated AMPK; P-mTOR, phosphorylated mTOR; VitD-def, Vitamin D deficient; VitD-nor, Vitamin D normal; VitD-sup, Vitamin D supplementation; TG, triglyceride; TC, total cholesterol; HDL-C, high-density lipoprotein cholesterol; LDL-C, low-density lipoprotein cholesterol; ALT, alanine transaminase; AST, aspartate aminotransferase
